# *Acinetobacter baumannii* Isolated from Lebanese Patients: Phenotypes and Genotypes of Resistance, Clonality, and Determinants of Pathogenicity

**DOI:** 10.3389/fcimb.2016.00163

**Published:** 2016-11-25

**Authors:** Elias Dahdouh, Micheline Hajjar, Monica Suarez, Ziad Daoud

**Affiliations:** ^1^Department of Animal Health, Faculty of Veterinary, Universidad Complutense de MadridMadrid, Spain; ^2^Department of Clinical Microbiology, Faculty of Medicine, University of BalamandBalamand, Lebanon

**Keywords:** *Acinetobacter baumannii*, carbapenem resistance, virulence, clonality, oxacillinases

## Abstract

**Introduction:** *Acinetobacter baumannii* is a nosocomial pathogen that usually affects critically ill patients. High mortality rates have been associated with MDR *A. baumannii* infections. Carbapenem resistance among these isolates is increasing worldwide and is associated with certain International Clones (ICs) and oxacillinases (OXAs). Moreover, this organism possesses a wide range of virulence factors, whose expression is not yet fully understood. In this study, clinical *A. baumannii* isolates are characterized in terms of antibiotic resistance, mechanisms of carbapenem resistance, clonality, and virulence.

**Materials and Methods:** *A. baumannii* clinical isolates (*n* = 90) where obtained from a tertiary care center in Beirut, Lebanon. API 20NE strips in addition to the amplification of *bla*_OXA−51−like_ were used for identification. Antibiotic susceptibility testing by disk diffusion was then performed in addition to PCRs for the detection of the most commonly disseminated carbapenemases. Clonality was determined by tri-locus PCR typing and doubling times were determined for isolates with varying susceptibility profiles. Biofilm formation, hemolysis, siderophore production, proteolytic activity, and surface motility was then determined for all the isolates. Statistical analysis was then performed for the determination of associations.

**Results and Discussion:** 81 (90%) of the isolates were resistant to carbapenems. These high rates are similar to other multi-center studies in the country suggesting the need of intervention on a national level. 74 (91.3%) of the carbapenem resistant isolates harbored *bla*_OXA−23−like_ including two that also harbored *bla*_OXA−24−like_. 88.9% of the *A. baumannii* isolates pertained to ICII and three other international clones were detected, showing the wide dissemination of clones into geographically distinct locations. Virulence profiles were highly diverse and no specific pattern was observed. Nevertheless, an association between motility, siderophore production, and biofilm formation was detected (*p* < 0.05).

**Conclusions:** A very high rate of carbapenem resistance was detected, showing the need for immediate intervention. IC II and OXA-23-like were the most disseminated, reflecting their international dissemination. No specific associations were made between virulence and resistance, but instead associations among certain virulence factors were found. Investigating a more clonally diverse pool of isolates could help in the determination of associations between virulence and resistance.

## Introduction

*Acinetobacter baumannii* is a nosocomial pathogen that could cause severe secondary infections among critically ill patients (Gordon and Wareham, 2010). This organism has a wide range of intrinsic resistance mechanisms and a heightened ability to acquire resistance to a broad range of antimicrobial agents (Peleg et al., 2012). Mortality rates among critically ill patients infected with Multi-Drug Resistant (MDR) *A. baumannii* are high, especially when improper empirical treatments are given (Ñamendys-Silva et al., 2015).

Carbapenems have been the treatment of choice for treating critically ill patients with MDR *A. baumannii* infections (Breilh et al., 2013). However, the increasing rates of Carbapenem Resistant *A. baumannii* (CRAB) isolates (Tärnberg et al., 2016) have limited their efficacy and increased mortality rates among infected patients (Lemos et al., 2014). Oxacillinases (OXAs) are the most commonly identified mechanism of carbapenem resistance among *A. baumannii* isolates. These OXAs include OXA-23-like, OXA-24-like, and OXA-58-like (Nowak and Paluchowska, 2016). *A. baumannii* also harbors the intrinsic *bla*_OXA−51−like_ in its genome. This OXA is highly conserved among *A. baumannii* strains (Turton et al., 2006a), but does not convey resistance to carbapenems unless associated with insertion sequences (Turton et al., 2006b). A few globally disseminated International Clones (ICs) have been found to cause most CRAB infections worldwide and have been associated with the presence of these OXAs (Karah et al., 2012).

A global surveillance program showed that Europe and the Mediterranean regions harbored the highest rate of MDR *A. baumannii* isolates (Flamm et al., 2016). Moreover, IC II was found to be widely disseminated among these countries (Di Popolo et al., 2011). This clone was also found to be largely disseminated in Lebanon (Rafei et al., 2014). Moreover, CRAB isolates were found to increase in prevalence in this country throughout the past decade (Hamouche and Sarkis, 2012). In 2012, 88% of 724 *A. baumannii* isolates recovered from various Lebanese hospitals were found to be resistant to imipenem (Hammoudi et al., 2015a). Although OXA-24-like (Hammoudi et al., 2015b) and OXA-58-like (Zarrilli et al., 2008) have been detected in this country, OXA-23-like seems to be the most prevalent among CRAB isolates (Rafei et al., 2015).

In addition to the various mechanisms of resistance detected among *A. baumannii* isolates, this organism can deferentially express various virulence factors. These factors include biofilm formation, surface motility, hemolysis on blood agars, siderophore production, and exoprotease activity; among others (Antunes et al., 2011). Whole genome sequencing and insertional mutagenesis led to the identifying of 28 genetic islands coding for genes predicted to be involved in virulence in this organism. Disruption of 6 of these islands did indeed result in avirulent isolates, as shown by infection models using *Caenorhabditis elegans* and *Dictyostelium discoideum* (Smith et al., 2007). Among the genes within these islands, genes coding for type IV secretion systems, which are associated with surface motility (Eijkelkamp et al., 2011a), are also found. Of note, *A. baumannii* was shown to produce different patterns of motility, depending on the choice (Difco Bacto or Eiken) or concentration of the agar (Clemmer et al., 2011). Also among the genes involved in virulence detected in *A. baumannii*, are genes coding for hemolysins/hemagglutinins (Smith et al., 2007). The expression of these genes was reported on blood agar plates and in liquid assays (Antunes et al., 2011). Exoprotease activity was also reported for *A. baumannii* strains in the previous study, after prediction of the presence of exoprotease genes in the *A. baumannii* genome (Antunes et al., 2011).

Siderophore production, mainly through the biosynthesis of acinetobactin, was also identified as a virulence determinant in *A. baumannii*. Successful biosynthesis of this siderophore was shown to be needed for the induction of apoptosis of epithelial cells. Moreover, impairment of siderophore production was shown to reduce the ability of *A. baumannii* to persist in and kill the host, as shown through infection models using *Galleria mellonella* larvae (Gaddy et al., 2012). Biofilm formation, though at different rates and patterns, has also been reported among *A. baumannii* clinical isolates (Dahdouh et al., 2016). Several loci in the *A. baumannii* genome have been implicated with the production of biofilms. One such locus is that coding poly-β-(1-6)-N-acetylglucosamine, an important component of biofilms, and a virulence factor that is involved in cell to cell adherence and protection from host defenses (Bentancor et al., 2012). Impairment of biofilm production, in addition to pili synthesis and motility, was shown to attenuate virulence in mammalian septicemia models (Cerqueira et al., 2014).

The relationship between virulence and antimicrobial resistance seems to be a highly complex one that is still not completely understood (Peleg et al., 2012). Importantly, the effect of harboring OXAs on virulence in *A. baumannii* is not well defined (Beceiro et al., 2013). The aim of this study is to characterize *A. baumannii* isolates obtained from a Lebanese hospital in terms of antibiotic susceptibility, carbapenemases harbored, clonality, and virulence determinants. Moreover, the relationship between virulence and carbapenem resistance will be explored. This would help in providing clinicians and infection control specialists with crucial data that would allow for the development of successful empirical treatments and infection control measures. Moreover, our findings could open the way for the exploitation of the interplay between virulence and resistance in the clinical setting.

## Materials and methods

### Bacterial strains

Ninety five non-repetitive *A. baumannii* clinical isolates were obtained from Saint George University—University Medical Center (SGH-UMC) over a period extending from June 2013 until August 2014. The isolates were obtained from various patient specimens that include sputum, pus, urine, and blood. The strains were identified using 20NE API strips (BioMérieux, France). The isolates that were not identified as *Acinetobacter calcoaceticus—A. baumannii* by the biochemical tests were not selected for further investigation. Amplification of the intrinsic *bla*_OXA−51−like_ gene by PCR was then performed in order to verify that the isolate is *A. baumannii* (Turton et al., 2006a). The isolates were then stored at −20°C in Luria-Bertani (LB) Broth supplemented with 20% glycerol until used.

### Antimicrobial susceptibility testing

The Kirby Bauer disk diffusion method was performed according to the CLSI guidelines (2014) in order to determine the Antibiotic Susceptibility Profiles (ASTs) of the isolates. The results were interpreted according to the cutoff values of the CLSI document M100-S24 (Clinical and Laboratory Standards Institute, 2014). Antimicrobial agents tested for were cefotaxime (30 μg), ceftazidime (30 μg), cefepime (30 μg), piperacillin/tazobactam (100/10 μg), meropenem (10 μg), imipenem (10 μg), trimethoprim/sulfamethoxazole (1.25/23.75 μg), ciprofloxacin (5 μg), gentamycin (10 μg), and colistin (10 μg). In order to verify resistance to colistin, *E*-tests (BioMérieux Mercy l'Etoile, France) and minimum inhibitory concentrations by broth microdilution were performed for isolates that showed narrow inhibition zones around the colistin disks.

### Polymerase chain reactions

PCRs for the detection of *bla*_OXA−51−like_ (Turton et al., 2006a); *bla*_OXA−23−like_, *bla*_OXA−24−like_, and *bla*_OXA−58−like_ (Mostachio et al., 2009); and *bla*_OXA−48_, *bla*_NDM_, and *bla*_KPC_ (Poirel et al., 2011) as previously described by the respective authors were performed. International lineage was determined using tri-locus PCR typing as described by Turton et al. (2007). The table summarized by Karah et al. (2012) was used in order to assign the international clonality of the tested isolates. For all PCR reactions, a commercial master mix was used (Qiagen, USA) and the primers, in addition to their respective annealing temperatures, are shown in Table 1. The PCR conditions were an initial elongation at 94°C for 3 min; followed by 30 cycles of 94°C for 45 s, the respective annealing temperature (Table 1) for 45 s, and 72°C for 1 min; and a final extension step at 72°C for 5 min. For the *bla*_OXA−48_, *bla*_NDM_, and *bla*_KPC_ genes, the number of cycles was increased to 36, and the annealing time was increased to 50 s (Poirel et al., 2011). Positive controls for the respective genes were used from previous studies (Moubareck et al., 2009; Hammoudi et al., 2015b).

**Table 1:** **Primers used in this study with their respective annealing temperatures**.

**Primer**	**Sequence**	**Annealing Temperature**	**References**
Oxa-51-like-F	5′-ATGAACATTAAAGCACTC-3′	46°C	Turton et al., 2006b
Oxa-51-like-R	5′-CTATAAAATACCTAATTGTTC-3′		
Oxa-23-like-F	5′-GATCGGATTGGAGAACCAGA-3′	53°C	Mostachio et al., 2009
Oxa-23-like-R	5′-ATTTCTGACCGCATTTCCAT-3′		
Oxa-24-like-F	5′-GGTTAGTTGGCCCCCTTAAA-3′	53°C	Mostachio et al., 2009
Oxa-24-like-R	5′-AGTTGAGCGAAAAGGGGATT-3′		
Oxa-58-like-F	5′-AAGTATTGGGGCTTGTGCTG-3′	53°C	Mostachio et al., 2009
Oxa-58-like-R	5′-CCCCTCTGCGCTCTACATAC-3′		
KPC-F	5′-CGTCTAGTTCTGCTGTCTTG-3′	52°C	Poirel et al., 2011
KPC-R	5′-CTTGTCATCCTTGTTAGGCG-3′		
NDM-F	5′-GGTTTGGCGATCTGGTTTTC-3′	52°C	Poirel et al., 2011
NDM-R	5′-CGGAATGGCTCATCACGATC-3′		
Oxa-48-F	5′-GCGTGGTTAAGGATGAACAC-3′	52°C	Poirel et al., 2011
Oxa-48-R	5′-CATCAAGTTCAACCCAACCG-3′		
Group1ompAF306	5′-GATGGCGTAAATCGTGGTA-3′	57°C	Turton et al., 2007
Group1and2ompAR660	5′-CAACTTTAGCGATTTCTGG-3′		
Group1csuEF	5′-CTTTAGCAAACATGACCTACC-3′	57°C	Turton et al., 2007
Group1csuER	5′-TACACCCGGGTTAATCGT-3′		
Gp1OXA66F89	5′-GCGCTTCAAAATCTGATGTA-3′	57°C	Turton et al., 2007
Gp1OXA66R647	5′-GCGTATATTTTGTTTCCATTC-3′		
Group2ompAF378	5′-GACCTTTCTTATCACAACGA-3′	57°C	Turton et al., 2007
Group1and2ompAR660	5′-CAACTTTAGCGATTTCTGG-3′		
Group2csuEF	5′-GGCGAACATGACCTATTT-3′	57°C	Turton et al., 2007
Group2csuER	5′-CTTCATGGCTCGTTGGTT-3′		
Gp2OXA69F169	5′-CATCAAGGTCAAACTCAA-3′	57°C	Turton et al., 2007
Gp2OXA69R330	5′-TAGCCTTTTTTCCCCATC-3′		

### Growth curves

In order to calculate the doubling time for selected isolates with varying antibiotic susceptibility profiles, 1:100 dilutions from overnight cultures liquid cultures in fresh LB broth were performed. The fresh suspensions were then incubated at 37°C with shaking at 200 rpm for 8 h. Each hour, the OD_600_ was measured and the doubling times were calculated from the resulting curve as previously described (Hall et al., 2014). The growth curves and all the following experiments were performed in triplicates.

### Biofilm formation

Biofilm formation was detected in polystyrene tubes after staining with crystal violet as previously described (Tomaras et al., 2003). Briefly, 1 mL of inoculated LB broth was incubated overnight at 37°C and then washed and stained with 1% crystal violet for 10 min. The dye was then rinsed away and biofilms were visualized and graded as “++” if heavy stains were observed and as “+” if faint stains were observed.

### Hemolytic activity

Hemolytic activity on blood agar plates were tested for by inoculating 10 μL of a bacterial suspension adjusted to 10^6^ CFU/mL in the center of these plates. The plates were then incubated for 6 days at 37°C and observed daily (Taybali et al., 2012).

### Siderophore production

Siderophore production in a liquid medium using the Chrome Azurol Solution (CAS) was performed as previously described (Louden et al., 2011). 5 mL of the PMS_7_-Ca medium was inoculated and incubated for 72 h. The suspension was then centrifuged at 4000 × g for 10 min and 1 mL of the filter-sterilized supernatant was incubated 1:1 with the CAS. The OD_630_ was then measured and a 10% difference between the sample and un-inoculated PMS_7_-Ca with CAS was considered as positive (Machuca and Milagres, 2003).

### Surface motility

For the detection of surface motility, a single colony was grown in LB broth overnight at 37°C. The visual turbidity of the suspension was then adjusted to be equivalent to the 0.5McFarland standard, in order to contain around 10^8^ CFU/mL. 1 μL (10^5^ CFU/mL) of the bacterial suspension was then inoculated on freshly prepared 0.3% LB-Agar (Difco, BD, USA) plates. The plates were then incubated at 37°C for 14 h and the diameter of the circular diffusion pattern was measured (Clemmer et al., 2011).

### Proteolytic activity

Proteolytic activity was determined by inoculating 5 mL of Trypticase Soy Broth Dyalisate with one colony and incubating it overnight at 37°C with shaking at 200 rpm. The following day, the suspension was centrifuged at 4000 × g for 10 min and 500 μL of the filter-sterilized supernatant was incubated with 500 μL of 1 mg/mL Azoalbumin dissolved in 50 mM Tris-HCl (pH = 7.7). This preparation was incubated at 37°C for 24 h and 13% trichloroacetic acid was then added. The tubes were incubated at −20°C for 20 min and centrifuged at 15,000 × g for 10 min. The OD_440_ of the supernatant was then measured and U/L values were calculated where one U was the amount of enzyme needed to degrade one micromole of Azoalbumin (Ronca-Testoni, 1983; Antunes et al., 2011)

### Statistical analysis

Normality of the data, when applicable, was tested for using the Shapiro-Wilk test. One-way ANOVA and student *t*-tests were performed for normally distributed data while the Kruskal-Wallis and Mann-Whitney tests were performed non-normally distributed data. Qualitative data was analyzed using the chi squared and the two-sided Fisher's exact tests. *P*-values of less than 0.05 were considered as statistically significant and all tests were performed using the SPSS program, version 17.0 (SPSS 111 Inc., Chicago, USA).

## Results

### Bacterial isolates

In total, 95 *Acinetobacter* spp. isolates were collected. Of these isolates, three were *Acinetobacter haemolyticus, one* was *Acinetobacter junii*/*johnsonii*, and one was *Acinetobacter radioresistens*/*lwoffii* as identified by the API strips. The rest of the isolates (*n* = 90) were *A. baumannii* as identified by API and the subsequent amplification of *bla*_*OXA*−51−*like*_ (Turton et al., 2006a). Thirty five (38.9%) *A. baumannii* isolates were collected in 2013 while fifty five (61.1%) were collected in 2014. Fifty eight (64.5%) of the *A. baumannii* isolates were collected from sputum, 17 (18.9%) from pus, 9 (10%) from urine, and 4 (4.4%) from blood samples. Two (2.2%) isolates were collected from catheters. Thirty six (40%) of the isolates were obtained from the ICU, 33 (36.7%) from general medicine, 10 (11.1%) from the surgical unit, 9 (10%) from the geriatric unit, 1 (1.1%) from the psychiatric unit, and 1 (1.1%) from the pediatric unit.

### Antibiotic susceptibility

Antibiotic susceptibility profiles were obtained after testing by the Kirby Bauer disk diffusion method according to CLSI guidelines (2014). Eighty one (90%) of the *A. baumannii* isolates were resistant to both meropenem and imipenem. Only one isolate was resistant to colistin. Susceptibility to other antimicrobial agents tested for was very low and did not exceed 14.4% (Table 2). Antibiotic susceptibility profiles are found in Supplementary Table 1.

**Table 2:** **Antibiotic susceptibility profiles for 90 ***A. baumannii*** isolates collected over a 1-year period**.

**ANTIMICROBIAL AGENT**										
	**CTX**		**CAZ**		**FEP**		**TZP**		**MEM**	
	**n**	**%**	**n**	**%**	**n**	**%**	**n**	**%**	**n**	**%**
Resistant	79	87.8	78	86.7	58	64.4	81	90	81	90
Intermediate	9	10	1	1.1	19	21.1	2	2.2	0	0
Sensitive	2	2.2	11	12.2	13	14.4	7	7.8	9	10
**ANTIMICROBIAL AGENT**										
	**IMP**		**SXT**		**CIP**		**GT**		**COL**	
	**n**	**%**	**n**	**%**	**n**	**%**	**n**	**%**	**n**	**%**
Resistant	81	90	83	92.2	84	93.3	77	85.6	1	1.1
Intermediate	0	0	0	0	0	0	0	0	0	0
Sensitive	9	10	7	7.8	6	6.7	13	14.4	89	98.9

*CTX stands for cefotaxime, CAZ for ceftazidime, FEP for cefepime, TZP for piperacillin/tazobactam, MEM for meropenem, IMP for imipenem, SXT for trimethoprim/sulfamethoxazole, CIP for ciprofloxacin, GT for gentamycin, and COL for colistin*.

### Dissemination of carbapenemases and international clones

PCRs for the detection of the most common carbapenemases among *A. baumannii* were performed in addition to tri-locus PCR typing which determines international clonality. Of the 81 *A. baumannii* isolates that showed resistance to carbapenems, 74 (91.3%) harbored *bla*_OXA−23−like_. Two of these isolates additionally harbored *bla*_OXA−24−like_. These are isolates 42 and 49. None of the other carbapenemases tested for were detected. Seven isolates were resistant to carbapenems but did not harbor any of the tested carbapenemases (Isolates 54, 59, 68, 72, 83, 84, and 85). Two isolates were sensitive to carbapenems but harbored *bla*_OXA−23−like_ (Isolates 17 and 87).

Eighty *A. baumannii* isolates (88.9%) pertained to IC II, whereas six (6.7%) pertained to group 4 (Isolates 11, 25, 39, 62, 82, and 91), one (1.1%) to group 10 (Isolate 56), and two (2.2%) to group 14 (Isolates 42 and 72), as summarized by Karah et al. (2012) (Table 3). One *A. baumannii* isolate showed a pattern that did not pertain to any IC where *CsuE* and *bla*_OXA−66_ were amplified from the first multiplex, and the other allele of *CsuE* was also amplified from the second multiplex.

**Table 3:** **Virulence determinants of 90 ***A. baumannii*** isolates in addition to international clonality and carbapenem resistance**.

**Isolate**	**IC**	**Carb R**.	**Bio**	**Hemo**	**Sidero**	**Motility (mm)**	**Proteo (U/L)**	**DT (hours)**
2	2	+	+	–	+	8±0.7	7.66±1.81	
3	2	+	++	–	+	7.5±0.7	25.32±4.22	
4	2	+	++	–	+	9.1±0.5	28.9±6.34	0.594±0.036
5	2	+	++	β/D1	+	18.1±2.8	61.97±12.65	
7	2	+	++	α/D3	+	8.6±1.2	23.68±10.74	
8	2	+	+	α/D2	+	9.1±1.1	20.56±7.32	0.471±0.067
10	2	+	++	α/D2	+	20.8±3.3	5.59±3.68	
11	4	–	++	–	+	6.4±0.5	21.15±2.06	
12	2	+	++	–	+	8.2±1	3.33±1.61	
13	2	+	+	α/D3	+	48.4±2.6	7.47±2.98	0.403±0.056
14	2	+	++	α/D2	+	8±0.9	26.22±1.86	
15	2	+	++	–	–	8.2±0.8	32.28±5.44	0.539±0.063
16	2	+	++	α/D3	+	14.2±2	23.48±1.96	
17	2	–	++	–	+	23.2±1.4	16±1.39	0.283±0.031
18	−	–	++	–	+	25±2.8	22.47±3.39	
19	2	+	++	–	+	12.8±0.6	23.24±4.1	
20	2	+	++	α/D2	+	23.3±1.7	30.69±6.94	
21	2	+	++	–	+	43.1±2.2	27.26±0.63	0.377±0.031
22	2	–	+	–	+	6.7±0.8	5.98±5.45	0.310±0.063
23	2	+	++	α/D2	+	6.8±0.7	24.71±3.38	
24	2	+	++	–	+	16.9±0.9	5.26±1.42	
25	4	+	++	α/D2	+	26.1±1.5	21.45±3.9	
26	2	+	++	–	+	27.8±1.5	8.72±0.3	
27	2	+	++	–	+	22.9±1.3	18.32±0.63	
28	2	+	++	α/D2	–	27.5±1.4	10.53±4.43	0.653±0.049
29	2	+	++	–	+	27.3±1.5	32.27±10.28	
30	2	+	++	–	+	18.8±1	7.08±3.31	
31	2	+	++	–	+	23.6±2.1	27.71±3.9	
32	2	+	++	–	–	30.3±2.3	23.77±1.77	
33	2	+	++	α/D2	+	37.7±1.9	19.04±2.15	
34	2	+	++	–	+	25.1±1.5	23.24±9.42	
35	2	+	++	–	+	24.4±1.6	26.51±1.86	
36	2	+	++	α/D2	+	41.5±2.3	7.49±4.58	
37	2	+	++	–	+	29.1±2.4	23.24±4.47	
38	2	+	++	–	+	25.6±1.4	10.59±3.46	
39	4	+	–	α/D3	–	10.3±1.7	6.9±2.4	
40	2	+	++	–	+	27.4±1.9	6.68±1.93	
41	2	+	++	α/D2	–	23.3±1.5	9.09±2.32	0.401±0.030
42	14	+	++	–	+	23.2±2.1	20.85±4.92	
43	2	+	+	α/D3	+	30.6±2.2	21.15±7.6	
44	2	+	++	–	–	32±2.4	19.48±2.78	0.467±0.039
45	2	+	++	–	+	28.9±2.3	4.72±2.17	
46	2	+	++	–	+	25.1±1.5	11.05±4.5	
47	2	+	++	α/D2	+	30.8±2.3	15.94±6.88	
48	2	+	++	α/D2	+	26±1.6	11.65±4.2	
49	2	+	++	–	+	19.2±0.9	11.72±4.99	0.343±0.120
51	2	+	++	–	+	22.4±1.5	24.43±2.73	
52	2	+	++	α/D2	+	26±2.2	9.43±3.22	
53	2	+	++	α/D2	+	23.6±2.4	11.4±4.09	
54	2	+	++	–	–	20.3±1.3	16.22±5.1	
55	2	+	++	–	+	20.8±1.3	30.47±10	
56	10	−	++	α/D3	+	31.5±2.1	11.64±2.02	
57	2	+	+	α/D2	–	21.8±1.1	25.32±2.73	
58	2	+	–	α/D1	–	11.1±0.6	24.49±4.55	
59	2	+	++	α/D2	–	22.1±1.2	13.81±4.49	
60	2	+	++	–	–	24.6±1.8	8.37±2.92	
61	2	+	++	α/D3	+	21.7±1.1	21.61±0.24	
62	4	–	++	α/D2	–	23.2±1.4	7.3±0.21	
63	2	+	++	α/D1	–	11.1±0.7	4.4±1.63	
64	2	–	+	α/D1	–	13.7±0.6	10.6±1.45	0.339±0.065
65	2	+	++	α/D2	–	25.2±1.9	7.38±3.81	
66	2	+	++	α/D2	–	27.1±2.2	27.41±2.87	0.369±0.021
67	2	+	++	α/D2	+	30.7±1.9	22.94±2.87	
68	2	+	++	–	–	30±2.2	22.64±7.44	
69	2	+	++	–	–	31.2±2.8	28.3±2.1	0.385±0.056
70	2	+	++	α/D2	–	32.3±1.7	22.05±6.94	
71	2	+	++	–	–	28.3±2	26.81±3.1	
72	14	+	++	α/D3	+	20.2±1.3	22.34±2.36	
73	2	+	++	α/D1	–	34.7±2.5	11.08±5.56	
74	2	+	++	α/D2	–	28.3±1.5	9.17±0.93	
75	2	+	++	α/D2	–	22.3±1.8	8.45±3.1	0.328±0.076
76	2	+	++	α/D3	+	24.6±1.8	27.41±4.03	0.311±0.021
77	2	+	++	–	+	23.1±5.9	12.02±1.96	
78	2	+	++	–	–	35.7±2	12.94±2.61	0.262±0.021
79	2	+	++	–	+	32.7±1.8	11.13±0.69	
80	2	+	+	α/D2	–	35.5±1.8	22.94±2.87	
81	2	+	++	α/D3	–	39.2±1.5	26.81±0.89	
82	4	+	–	–	–	11.1±0.7	8.28±0.33	
83	2	+	+	–	–	28.6±2.4	20.85±4.5	
84	2	+	++	–	–	28.9±2.3	3.21±3.04	
85	2	+	++	–	–	47.8±2.6	8.49±2.85	
86	2	+	++	–	–	24.6±1.5	6.54±4.7	
87	2	–	++	–	+	23.4±1.9	13.9±4.48	
89	2	+	++	–	–	41.5±2.3	21.81±8.09	
90	2	+	++	α/D2	–	47.9±2.9	23.56±10.31	
91	4	+	+	α/D3	–	16.2±1	25.14±2.31	
92	2	+	++	–	–	41.4±2.1	6.04±3.49	
93	2	+	++	α/D3	–	44.3±2.5	24.55±9.35	
94	2	–	++	α/D2	–	144.5±7.7	26.37±4.42	
95	2	+	++	–	–	46.6±2.7	23.65±8.61	0.414±0.025

*IC stands for international clone, Carb R. for carbapenem resistance, Bio for biofilm production, Hemo for hemolysis where the type of hemolysis and the day (D1–D3) on which it was first observed was recorded, Sidero for siderophore production, Proteo for proteolytic activity, and DT for doubling times*.

### Virulence determinants in relation with clonality and carbapenem susceptibility

The result of the various virulence determinants, in addition to international clonality and carbapenem resistance, are shown in Table 3. Seventy seven (85.6%) of the *A. baumannii* isolates showed strong biofilm formations while 10 (11.1%) showed weak formations and 3 (3.3%) showed no biofilm formation. Forty two (46.7%) of the *A. baumannii* isolates showed α-hemolysis on blood agar plates while one isolate showed β-hemolysis. Seventy two (80%) *A. baumannii* isolates showed a diffusion pattern indicating surface motility on 0.3% LB-Agar while 52 (57.8%) isolates were positive for siderophore production. Proteolytic activity ranged from 4.4 ± 1.63 U/L to 61.97 ± 12.65 U/L and the doubling times for selected isolates ranged from 0.262 ± 0.021 to 0.653 ± 0.049 h.

No general pattern was observed between the doubling times and antibiotic susceptibility profiles. The slowest doubling time was determined for isolate 28 which was carbapenem-resistant, α-hemolytic, produced strong biofilms, showed a motility pattern, harbored *bla*_OXA−23−like_, and pertained to IC II. Nevertheless, other isolates with similar profiles showed faster doubling times (Table 3). One such example is isolate 78 which incidentally had the fastest doubling time. The colistin-resistant isolate 75 showed a similar antibiotic susceptibility pattern and virulence profile to the aforementioned two isolates and had a relatively fast doubling time of 0.328 ± 0.076 h.

**Figure 1:**
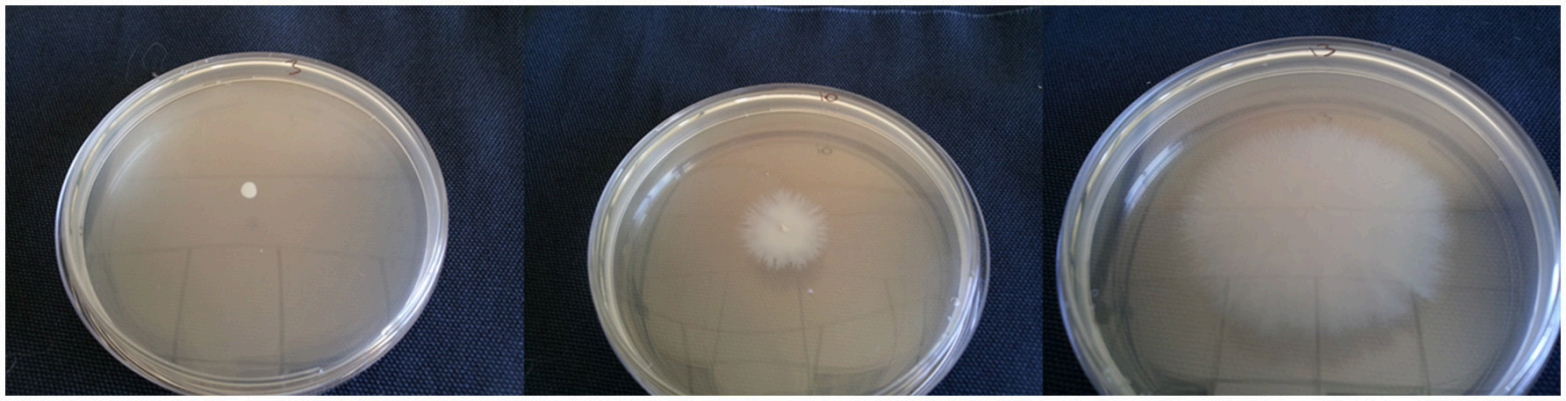
**Representative isolates showing (from left to right) no motility, moderate motility, and high motility**.

Figure 1 shows representative isolates for non-motile, moderately motile, and highly motile isolates. Isolate 94 showed the highest motility diffusion diameter (144.5 ± 7.7 mm on a 15 cm square petri dish), produced strong biofilms, showed hemolysis on blood agars, and had a relatively high proteolytic activity (26.37 ± 4.42 U/L). This isolate was susceptible to carbapenems and pertained to IC II. Isolate 5 was the only isolate that showed β-hemolysis and it had the highest proteolytic activity. It also showed a motility diffusion pattern, produced strong biofilms and siderophores, pertained to IC II, and harbored *bla*_OXA−23−like_. Isolates 2, 3, 4, 11, 12, and 82 showed very modest to no motility diffusion patterns and were negative for hemolysis. Isolate 4 also had a slow doubling time (0.594 ± 0.036 h). However, not all isolates that had similar motility diffusion diameters shared the rest of the virulence profile with these isolates.

Two of the isolates that pertained to group 4 were susceptible to carbapenems and four out of six isolates of this group were negative for siderophore production. Moreover, another two isolates of this group were negative for biofilm formation. Four of the six isolates pertaining to this group harbored *bla*_OXA−23−like_ and were resistant to carbapenems. The isolate pertaining to group ten was susceptible to carbapenems and showed elevated levels of virulence determinants. Both isolates pertaining to Group 14 had similar profiles but one was negative for *bla*_OXA−23−like_ but positive for hemolysis whereas the other harbored *bla*_OXA−23−like_ and *bla*_OXA−24−like_ but was negative for hemolysis. Isolate 18, which did not pertain to any of the international clones by tri-locus sequence typing was susceptible to carbapenems and had similar virulence profiles as isolates pertaining to IC II. Both isolates that harbored *bla*_OXA−24−like_ in addition to *bla*_OXA−23−like_ were negative for hemolysis and had similar virulence profiles.

### Associations between virulence and resistance

IC II was positively associated with both carbapenem resistance and harboring *bla*_OXA−23−like_ (*p* < 0.01). All the isolates that did not produce biofilms were also negative for siderophore production. The isolates that showed moderate motility diffusion patterns were associated with strong biofilm formation (*p* < 0.01) while those that were either highly motile or non-motile showed a positive association with siderophore production (*p* < 0.05). No other statistical association were made.

## Discussion

In this study, *A. baumannii* isolates obtained from a major tertiary care center in Beirut, Lebanon were characterized in terms of antibiotic susceptibility, clonality, and virulence determinants. An extremely high rate of carbapenem resistance (90%) was detected among the *A. baumannii* isolates. This rate, however, is very similar to that reported from a nation-wide study (Hammoudi et al., 2015a) where the prevalence of CRAB isolates was 88%. These findings suggest an immediate need for the implementation of effective infection control measures and antibiotic stewardship programs in Lebanese hospitals. This need is even more urgent due to the high resistance rates of these isolates to other antimicrobial agents that were tested for in this study (Table 2). The low rate of resistance to colistin among these isolates hold a viable alternative for treatment. Nevertheless, its nephrotoxic effects (Bergen et al., 2012) and the ability of *A. baumannii* to develop resistance toward this antimicrobial agent during therapy (Valencia et al., 2009) limits its effectiveness.

In accordance with other studies performed in Lebanon, IC II was by far the most prevalent clone among the *A. baumannii* isolates (Rafei et al., 2014) and OXA-23-like is the most disseminated (Rafei et al., 2015). These findings are also similar to those reported from other Mediterranean countries (Di Popolo et al., 2011). Interestingly, although an outbreak caused by *bla*_OXA−58−like_ harboring CRAB isolates was reported from SGH-UMC a few years ago (Zarrilli et al., 2008), this carbapenemase was not detected among our isolates. This could be an indication of the successful eradication of the clone that caused the outbreak at the time of that study.

The presence of *bla*_OXA−23−like_ in two carbapenem-sensitive isolates suggest that either the expression of this gene in these isolates is very modest or that it harbors a mutation that renders it ineffective. Further characterization of these isolates by sequencing the genetic environment of *bla*_OXA−23−like_ and preforming RT-PCRs could help in better understanding why no carbapenem resistance was detected in these isolates. The seven CRAB isolates in which no carbapenemase was detected could be expressing resistance through carbapenemases that were not tested for in this study and/or through alteration of membrane permeability and efflux pump over-expression (Peleg et al., 2008).

Ten CRAB isolates pertaining to PCR group 4 were first identified in a study investigating *A. baumannii* isolates from several European countries (Towner et al., 2008). In our study, two out of the six isolates pertaining to this group were susceptible to carbapenems. Similarly, the isolate pertaining to group 10 was sensitive to carbapenems as opposed to the detection of carbapenem resistance among isolates pertaining to this group in a study in Portugal (Grosso et al., 2011). The two isolates pertaining to group 14 were both resistant to carbapenems. One of them did not harbor any of the tested carbapenemases while the other had both OXA-23-like and OXA-24-like. This group was first identified in a study from Romanian hospitals and the isolate pertaining to this group harbored *bla*_OXA−58−like_ (Bonnin et al., 2011). The diversity of profiles between the isolates pertaining to these groups, in addition to the diversity seen among isolates pertaining to IC II, reflect the plasticity of the *A. baumannii* genome (Antunes et al., 2014). Moreover, the presence of these clones, in addition to the presence of the globally disseminated IC II (Karah et al., 2012), show the global expansion of *A. baumannii* clones that are able to expand to wide geographical areas.

A study by Antunes et al. (2011) showed that different *A. baumannii* clinical isolates are able to display different virulence profiles. This was indeed shown to be the case in our study where no specific pattern of virulence was associated with a specific clone (Table 3). The *A. baumannii* isolates investigated had a high degree of variability in terms of virulence profiles. Moreover, no associations between antibiotic susceptibility profiles and doubling times were detected. These findings suggest that each isolate should be treated as a unique case and no general assumptions could be made based on clonality and AST. This also shows that the relationship between virulence and antibiotic resistance is indeed a complex one and warrants further investigation (Peleg et al., 2012). Nevertheless, the low diversity of clones among the tested isolates could be obscuring associations between clonality and virulence, since some isolates pertaining to group 4 and those pertaining to group 14 had similar virulence profiles. Further investigating these associations using larger and more clonally diversified pools could shed further light on the matter. Interestingly, the motility diffusion patterns that were obtained in our study using Difco agar were circular, as opposed to the linear patterns reported while using this kind of agar by Clemmer et al. (2011). The patterns obtained in our study are rather similar to those obtained using Eiken agar. The reason for this difference is still not clear at this point, but could possibly be due to an experimental factor that is independent from the agar brand, or is a property pertaining to the particular strains that were tested for in the different studies. This, however, requires further investigation before verification.

Finally, while comparing virulence factors one to another, an association between motility on one hand, and biofilm formation and siderophore production on the other, was determined. The relationship between motility and strong biofilm formation has been previously reported among MDR *A. baumannii* isolates (Eijkelkamp et al., 2011b). Moreover, the association detected between motility and siderophore production is not surprising since the former factor was associated with biofilm production while the latter allows for iron acquisition that is crucial for biofilm formation (Gentile et al., 2014). These associations reveal a highly complex interplay between the different virulence determinants in *A. baumannii*, especially those that are multi-factorial.

## Conclusions

In conclusion, a very high rate of carbapenem resistance was detected among clinical *A. baumannii* isolates obtained from a Lebanese tertiary care center. IC II was the most prevalent clone and OXA-23-like was the most prevalent carbapenemase. The isolates showed highly varied virulence profiles that were not associated with any specific clone or oxacillinase gene. However, associations between motility, biofilm formation, and siderophore production have been found. Increasing the diversity of the pool of isolates could reveal associations between clonality and virulence that could allow for the prediction of pathogenicity of a clinical *A. baumannii* isolate.

## Author contributions

ED: performed the virulence experiments, clonality analysis, part of the PCRs, and statistical analysis. Was also involved in experiment design and data analysis, and drafted the manuscript. MH: performed antibiotic susceptibility analysis and part of the PCRs, obtained the isolates that were included in this work, and was involve in the manuscript preparation. MS: was involved in study design, data analysis, and revision of the manuscript. ZD: was involved in study design, data analysis, revision of the manuscript, and supervised all the work that was done at his laboratory. All authors have approved the final version of the manuscript and are accountable for its content.

### Conflict of interest statement

The authors declare that the research was conducted in the absence of any commercial or financial relationships that could be construed as a potential conflict of interest.
