# Supplementary material for: Acinetobacter baumannii Isolated from Lebanese Patients: Phenotypes and Genotypes of Resistance, Clonality, and Determinants of Pathogenicity
Source: Front Cell Infect Microbiol. 2016 Nov 25;6:163. doi: 10.3389/fcimb.2016.00163 (PMC5122575; doi:10.3389/fcimb.2016.00163)
Supplement: Supplementary file 1 [file Table1.docx]

Supplementary Material

***Acinetobacter baumannii* Isolated from Lebanese Patients: Phenotypes and Genotypes of Resistance, Clonality, and Determinants of Pathogenicity**

**Elias Dahdouh^1^, Micheline Hajjar^2^, Monica Suarez^1^, and Ziad Daoud^2*^.**

*** Correspondence:** Ziad Daoud: ziad.daoud@balamand.edu.lb

**Supplementary Table 1.** Inhibition zone diameters for the tested antimicrobial agents for 90 *A. baumannii* isolates collected over a one-year period. CTX stands for cefotaxime, CAZ for ceftazidime, FEP for cefepime, TZP for piperacillin/tazobactam, MEM for meropenem, IMP for imipenem, SXT for trimethoprim/sulfamethoxazole, CIP for ciprofloxacin, GT for gentamycin, and COL for colistin. The * indicates that the results were validated by the determination of the MIC. S stands for susceptible, I for Intermediately resistance, and R for Resistant.

|  | **Antibiotic Susceptibility Testing** | | | | | | | | | | | | | | | | | | | | **Carbapenemases** | |
| --- | --- | --- | --- | --- | --- | --- | --- | --- | --- | --- | --- | --- | --- | --- | --- | --- | --- | --- | --- | --- | --- | --- |
|  | **CTX** | | **CAZ** | | **FEP** | | **TZP** | | **MEM** | | **IMP** | | **SXT** | | **CIP** | | **GT** | | **COL*** | | **OXA-23-like** | **OXA-24-like** |
| **Isolate** | **mm** | **S-I-R** | **mm** | **S-I-R** | **mm** | **S-I-R** | **mm** | **S-I-R** | **mm** | **S-I-R** | **mm** | **S-I-R** | **mm** | **S-I-R** | **mm** | **S-I-R** | **mm** | **S-I-R** | **mm** | **S-I-R** |  |  |
| 2 | 6 | R | 6 | R | 10 | R | 6 | R | 6 | R | 6 | R | 6 | R | 6 | R | 6 | R | 12 | S | + | - |
| 3 | 6 | R | 6 | R | 6 | R | 6 | R | 6 | R | 8 | R | 6 | R | 6 | R | 6 | R | 12 | S | + | - |
| 4 | 6 | R | 6 | R | 10 | R | 6 | R | 6 | R | 6 | R | 6 | R | 6 | R | 6 | R | 12 | S | + | - |
| 5 | 6 | R | 6 | R | 15 | I | 9 | R | 12 | R | 15 | R | 6 | R | 6 | R | 6 | R | 13 | S | + | - |
| 7 | 6 | R | 6 | R | 10 | R | 6 | R | 6 | R | 6 | R | 6 | R | 6 | R | 15 | S | 18 | S | + | - |
| 8 | 6 | R | 6 | R | 12 | R | 8 | R | 6 | R | 6 | R | 6 | R | 6 | R | 6 | R | 11 | S | + | - |
| 10 | 15 | I | 19 | S | 27 | S | 17 | R | 11 | R | 17 | R | 20 | S | 22 | S | 20 | S | 11 | S | + | - |
| 11 | 17 | I | 20 | S | 21 | S | 21 | S | 21 | S | 26 | S | 14 | S | 21 | S | 17 | S | 12 | S | - | - |
| 12 | 6 | R | 6 | R | 11 | R | 6 | R | 6 | R | 8 | R | 6 | R | 6 | R | 6 | R | 11 | S | + | - |
| 13 | 6 | R | 6 | R | 12 | R | 6 | R | 6 | R | 6 | R | 6 | R | 6 | R | 6 | R | 11 | S | + | - |
| 14 | 6 | R | 6 | R | 10 | R | 6 | R | 6 | R | 10 | R | 6 | R | 6 | R | 6 | R | 13 | S | + | - |
| 15 | 6 | R | 6 | R | 6 | R | 6 | R | 6 | R | 6 | R | 6 | R | 6 | R | 6 | R | 12 | S | + | - |
| 16 | 6 | R | 6 | R | 11 | R | 7 | R | 6 | R | 6 | R | 6 | R | 6 | R | 6 | R | 13 | S | + | - |
| 17 | 13 | R | 17 | I | 19 | S | 18 | I | 18 | S | 24 | S | 6 | R | 6 | R | 6 | R | 11 | S | + | - |
| 18 | 16 | I | 19 | S | 20 | S | 20 | S | 23 | S | 28 | S | 6 | R | 6 | R | 6 | R | 13 | S | - | - |
| 19 | 6 | R | 6 | R | 12 | R | 9 | R | 10 | R | 11 | R | 6 | R | 6 | R | 6 | R | 11 | S | + | - |
| 20 | 6 | R | 6 | R | 14 | R | 6 | R | 10 | R | 10 | R | 6 | R | 6 | R | 6 | R | 13 | S | + | - |
| 21 | 6 | R | 6 | R | 12 | R | 8 | R | 8 | R | 11 | R | 6 | R | 6 | R | 6 | R | 12 | S | + | - |
| 22 | 16 | I | 18 | S | 21 | S | 19 | I | 21 | S | 28 | S | 20 | S | 24 | S | 17 | S | 13 | S | - | - |
| 23 | 6 | R | 6 | R | 13 | R | 12 | R | 8 | R | 8 | R | 6 | R | 6 | R | 6 | R | 13 | S | + | - |
| 24 | 6 | R | 6 | R | 13 | R | 6 | R | 12 | R | 12 | R | 6 | R | 6 | R | 6 | R | 13 | S | + | - |
| 25 | 6 | R | 6 | R | 14 | R | 13 | R | 6 | R | 12 | R | 6 | R | 6 | R | 6 | R | 12 | S | + | - |
| 26 | 6 | R | 6 | R | 13 | R | 10 | R | 6 | R | 11 | R | 6 | R | 6 | R | 6 | R | 12 | S | + | - |
| 27 | 6 | R | 6 | R | 11 | R | 6 | R | 6 | R | 6 | R | 6 | R | 6 | R | 6 | R | 12 | S | + | - |
| 28 | 6 | R | 6 | R | 10 | R | 6 | R | 6 | R | 6 | R | 6 | R | 6 | R | 6 | R | 14 | S | + | - |
| 29 | 6 | R | 6 | R | 11 | R | 9 | R | 6 | R | 9 | R | 6 | R | 6 | R | 6 | R | 13 | S | + | - |
| 30 | 6 | R | 6 | R | 6 | R | 6 | R | 6 | R | 6 | R | 6 | R | 6 | R | 6 | R | 13 | S | + | - |
| 31 | 6 | R | 6 | R | 9 | R | 6 | R | 6 | R | 6 | R | 6 | R | 6 | R | 6 | R | 15 | S | + | - |
| 32 | 6 | R | 6 | R | 11 | R | 6 | R | 6 | R | 9 | R | 6 | R | 6 | R | 6 | R | 11 | S | + | - |
| 33 | 6 | R | 6 | R | 10 | R | 6 | R | 6 | R | 6 | R | 6 | R | 6 | R | 6 | R | 12 | S | + | - |
| 34 | 6 | R | 6 | R | 16 | I | 6 | R | 11 | R | 13 | R | 6 | R | 6 | R | 6 | R | 12 | S | + | - |
| 35 | 6 | R | 6 | R | 14 | R | 6 | R | 6 | R | 6 | R | 6 | R | 6 | R | 6 | R | 12 | S | + | - |
| 36 | 6 | R | 6 | R | 14 | R | 9 | R | 8 | R | 13 | R | 6 | R | 6 | R | 6 | R | 13 | S | + | - |
| 37 | 6 | R | 6 | R | 13 | R | 6 | R | 6 | R | 10 | R | 6 | R | 6 | R | 6 | R | 11 | S | + | - |
| 38 | 6 | R | 6 | R | 14 | R | 9 | R | 10 | R | 6 | R | 6 | R | 6 | R | 6 | R | 13 | S | + | - |
| 39 | 6 | R | 6 | R | 13 | R | 6 | R | 6 | R | 6 | R | 6 | R | 6 | R | 15 | S | 10 | S | + | - |
| 40 | 6 | R | 6 | R | 10 | R | 6 | R | 6 | R | 8 | R | 6 | R | 6 | R | 6 | R | 12 | S | + | - |
| 41 | 6 | R | 6 | R | 13 | R | 9 | R | 6 | R | 10 | R | 6 | R | 6 | R | 6 | R | 13 | S | + | - |
| 42 | 15 | I | 23 | S | 25 | S | 13 | R | 6 | R | 6 | R | 6 | R | 6 | R | 6 | R | 16 | S | + | + |
| 43 | 6 | R | 6 | R | 14 | R | 9 | R | 14 | R | 15 | R | 6 | R | 6 | R | 6 | R | 12 | S | + | - |
| 44 | 6 | R | 6 | R | 14 | R | 11 | R | 10 | R | 13 | R | 6 | R | 6 | R | 6 | R | 13 | S | + | - |
| 45 | 6 | R | 6 | R | 6 | R | 6 | R | 6 | R | 12 | R | 6 | R | 6 | R | 6 | R | 14 | S | + | - |
| 46 | 6 | R | 6 | R | 15 | I | 6 | R | 8 | R | 13 | R | 6 | R | 6 | R | 6 | R | 11 | S | + | - |
| 47 | 6 | R | 6 | R | 16 | I | 12 | R | 10 | R | 13 | R | 6 | R | 6 | R | 6 | R | 12 | S | + | - |
| 48 | 6 | R | 6 | R | 14 | R | 10 | R | 11 | R | 12 | R | 6 | R | 6 | R | 6 | R | 12 | S | + | - |
| 49 | 16 | I | 21 | S | 23 | S | 15 | R | 6 | R | 10 | R | 6 | R | 6 | R | 6 | R | 13 | S | + | + |
| 51 | 6 | R | 6 | R | 17 | I | 10 | R | 6 | R | 12 | R | 6 | R | 6 | R | 6 | R | 12 | S | + | - |
| 52 | 6 | R | 6 | R | 11 | R | 8 | R | 9 | R | 11 | R | 13 | R | 6 | R | 6 | R | 14 | S | + | - |
| 53 | 6 | R | 6 | R | 14 | R | 12 | R | 11 | R | 13 | R | 6 | R | 6 | R | 6 | R | 12 | S | + | - |
| 54 | 6 | R | 6 | R | 11 | R | 8 | R | 10 | R | 13 | R | 6 | R | 6 | R | 6 | R | 13 | S | - | - |
| 55 | 6 | R | 6 | R | 12 | R | 8 | R | 8 | R | 10 | R | 6 | R | 6 | R | 6 | R | 11 | S | + | - |
| 56 | 17 | I | 20 | S | 23 | S | 21 | S | 23 | S | 36 | S | 20 | S | 28 | S | 19 | S | 11 | S | - | - |
| 57 | 6 | R | 6 | R | 16 | I | 12 | R | 12 | R | 13 | R | 6 | R | 6 | R | 6 | R | 12 | S | + | - |
| 58 | 6 | R | 6 | R | 16 | I | 11 | R | 11 | R | 16 | R | 6 | R | 6 | R | 16 | S | 10 | S | + | - |
| 59 | 6 | R | 6 | R | 15 | I | 6 | R | 6 | R | 10 | R | 6 | R | 6 | R | 6 | R | 10 | S | - | - |
| 60 | 6 | R | 6 | R | 14 | R | 13 | R | 10 | R | 12 | R | 6 | R | 6 | R | 6 | R | 14 | S | + | - |
| 61 | 6 | R | 6 | R | 15 | I | 12 | R | 12 | R | 13 | R | 6 | R | 6 | R | 6 | R | 12 | S | + | - |
| 62 | 6 | R | 10 | R | 18 | S | 11 | R | 23 | S | 30 | S | 6 | R | 6 | R | 6 | R | 12 | S | - | - |
| 63 | 6 | R | 6 | R | 12 | R | 10 | R | 13 | R | 16 | R | 6 | R | 6 | R | 6 | R | 13 | S | + | - |
| 64 | 18 | I | 24 | S | 26 | S | 22 | S | 27 | S | 30 | S | 22 | S | 6 | R | 23 | S | 13 | S | - | - |
| 65 | 6 | R | 6 | R | 11 | R | 10 | R | 9 | R | 12 | R | 6 | R | 6 | R | 6 | R | 12 | S | + | - |
| 66 | 6 | R | 6 | R | 14 | R | 11 | R | 12 | R | 13 | R | 6 | R | 6 | R | 6 | R | 13 | S | + | - |
| 67 | 6 | R | 6 | R | 11 | R | 6 | R | 6 | R | 11 | R | 6 | R | 6 | R | 6 | R | 13 | S | + | - |
| 68 | 6 | R | 6 | R | 8 | R | 6 | R | 6 | R | 10 | R | 6 | R | 6 | R | 6 | R | 13 | S | - | - |
| 69 | 6 | R | 6 | R | 15 | I | 12 | R | 6 | R | 10 | R | 6 | R | 6 | R | 18 | S | 14 | S | + | - |
| 70 | 6 | R | 6 | R | 12 | R | 10 | R | 8 | R | 12 | R | 6 | R | 6 | R | 6 | R | 12 | S | + | - |
| 71 | 6 | R | 6 | R | 15 | I | 10 | R | 11 | R | 11 | R | 6 | R | 6 | R | 6 | R | 13 | S | + | - |
| 72 | 18 | I | 22 | S | 24 | S | 15 | R | 6 | R | 6 | R | 6 | R | 6 | R | 6 | R | 14 | S | - | - |
| 73 | 6 | R | 6 | R | 13 | R | 9 | R | 6 | R | 9 | R | 6 | R | 6 | R | 6 | R | 12 | S | + | - |
| 74 | 6 | R | 6 | R | 12 | R | 6 | R | 10 | R | 11 | R | 6 | R | 6 | R | 6 | R | 14 | S | + | - |
| 75 | 6 | R | 6 | R | 15 | I | 11 | R | 10 | R | 14 | R | 10 | R | 6 | R | 6 | R | 6 | R | + | - |
| 76 | 6 | R | 6 | R | 15 | I | 11 | R | 11 | R | 13 | R | 6 | R | 6 | R | 6 | R | 12 | S | + | - |
| 77 | 6 | R | 6 | R | 13 | R | 8 | R | 8 | R | 12 | R | 6 | R | 6 | R | 6 | R | 12 | S | + | - |
| 78 | 6 | R | 6 | R | 15 | I | 8 | R | 6 | R | 12 | R | 6 | R | 6 | R | 6 | R | 13 | S | + | - |
| 79 | 6 | R | 6 | R | 15 | I | 7 | R | 7 | R | 12 | R | 6 | R | 6 | R | 6 | R | 13 | S | + | - |
| 80 | 6 | R | 6 | R | 17 | I | 16 | R | 10 | R | 13 | R | 6 | R | 6 | R | 6 | R | 12 | S | + | - |
| 81 | 6 | R | 6 | R | 15 | I | 12 | R | 11 | R | 12 | R | 6 | R | 6 | R | 6 | R | 12 | S | + | - |
| 82 | 6 | R | 6 | R | 11 | R | 6 | R | 10 | R | 11 | R | 6 | R | 6 | R | 15 | S | 12 | S | + | - |
| 83 | 6 | R | 6 | R | 15 | I | 9 | R | 6 | R | 14 | R | 6 | R | 6 | R | 6 | R | 12 | S | - | - |
| 84 | 6 | R | 6 | R | 14 | R | 7 | R | 6 | R | 11 | R | 6 | R | 6 | R | 6 | R | 13 | S | - | - |
| 85 | 6 | R | 6 | R | 12 | R | 9 | R | 9 | R | 11 | R | 6 | R | 6 | R | 6 | R | 12 | S | - | - |
| 86 | 6 | R | 6 | R | 14 | R | 6 | R | 11 | R | 13 | R | 6 | R | 6 | R | 6 | R | 14 | S | + | - |
| 87 | 20 | S | 24 | S | 27 | S | 24 | S | 30 | S | 38 | S | 16 | S | 27 | S | 21 | S | 12 | S | + | - |
| 89 | 6 | R | 6 | R | 9 | R | 6 | R | 6 | R | 6 | R | 6 | R | 6 | R | 6 | R | 11 | S | + | - |
| 90 | 6 | R | 6 | R | 10 | R | 6 | R | 6 | R | 6 | R | 6 | R | 6 | R | 6 | R | 12 | S | + | - |
| 91 | 6 | R | 6 | R | 13 | R | 6 | R | 6 | R | 12 | R | 6 | R | 6 | R | 18 | S | 11 | S | + | - |
| 92 | 6 | R | 6 | R | 15 | I | 6 | R | 11 | R | 11 | R | 6 | R | 6 | R | 6 | R | 15 | S | + | - |
| 93 | 6 | R | 6 | R | 8 | R | 6 | R | 6 | R | 7 | R | 6 | R | 6 | R | 6 | R | 13 | S | + | - |
| 94 | 20 | S | 22 | S | 24 | S | 22 | S | 28 | S | 32 | S | 27 | S | 26 | S | 22 | S | 15 | S | - | - |
| 95 | 6 | R | 6 | R | 11 | R | 6 | R | 6 | R | 10 | R | 6 | R | 6 | R | 6 | R | 15 | S | + | - |

**
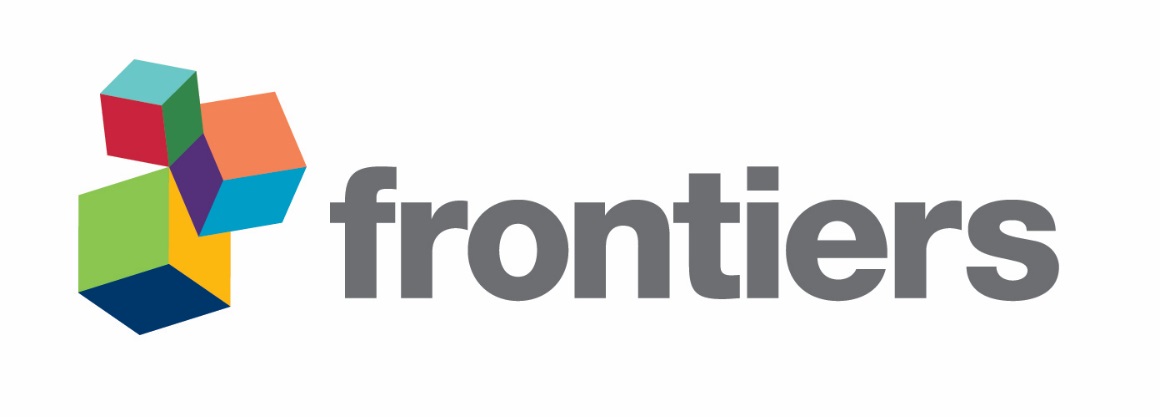
**
